# Adenoid cystic carcinoma in the maxillary gingiva: a case report and immunohistochemical study

**DOI:** 10.7497/j.issn.2095-3941.2013.01.009

**Published:** 2013-03

**Authors:** Chi Zhao, Jin-Zhong Liu, Shu-Bin Wang, Shi-Chang Wang

**Affiliations:** 1 Zhengzhou University School and Hospital of Stomatology, Zhengzhou 450000, China;; 2 Department of Pathology, The Fourth Affiliated Hospital of Zhengzhou University, Zhengzhou 450044, China; 3 Department of Oral and Maxillofacial Surgery, The Fourth Affiliated Hospital of Zhengzhou University, Zhengzhou 450044, China

**Keywords:** Gingival, maxilla, adenoid cystic carcinoma, immunohistochemistry

## Abstract

Gingival adenoid cystic carcinoma (ACC) is a rare malignancy. We describe the diagnosis and treatment of a 43 year-old woman who presented with a persistent oral ulcer for approximately 1 year, and subsequent pain in the left posterior maxillary region. Clinical examination revealed an ulcer in the left upper molar gingiva, with swelling in the region from the second premolar to the third molar. X-ray images demonstrated the involvement of the maxillary alveolar bone. The histopathological and immunohistochemical features were diagnostic of ACC. ACC is often presented as a gingival lesion; thus, it may easily be neglected by patients. The identification of this tumor using specific pathological analyses prevents misdiagnosis and enables clinicians to determine the appropriate treatment. In this case, no recurrence or distant metastasis was observed after 2 years of follow-up.

## Introduction

Adenoid cystic carcinoma (ACC) is an uncommon malignant neoplasm that develops within secretory glands. ACC commonly occurs in the major and minor salivary glands of the head and neck. Other sites include the lungs, breasts, lacrimal glands, and skin^1^. Given that the palate, oral floor, and buccal mucosa are commonly affected, dentists are generally familiar with ACC. However, gingival ACC is less common.

ACC is known to have long benign periods followed by instances of malignancy, but it may behave aggressively in some patients^2^. Thus, the course of ACC is unpredictable. The identification of this tumor using specific pathological analyses prevents misdiagnosis and aids clinicians to determine the appropriate treatment. Long-term follow-up is essential regardless of the site because ACC is prone to undergo late recurrence and metastasis^3^^,^^4^.

## Case report

**Figure 1:**
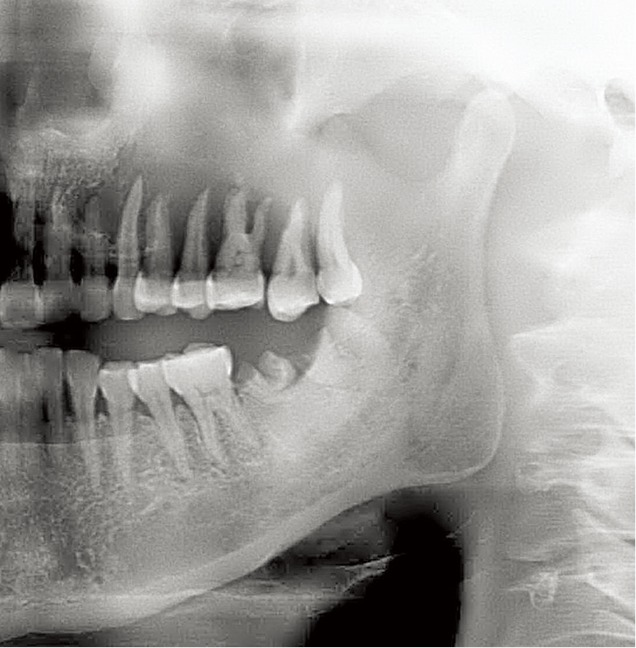
Panorex radiograph of a poorly defined, irregular osteolytic ACC lesion involving the left maxilla.

A 43 year-old woman complained of an oral ulcer that persisted for almost 1 year as well as pain in the left posterior maxillary region for 1 month. Clinical examination revealed an ulcer on the left upper molar gingiva, with swelling from the second premolar to the third molar in the same region. X-ray images showed the involvement of the maxillary alveolar bone (**Figure 1**).

**Figure 2:**
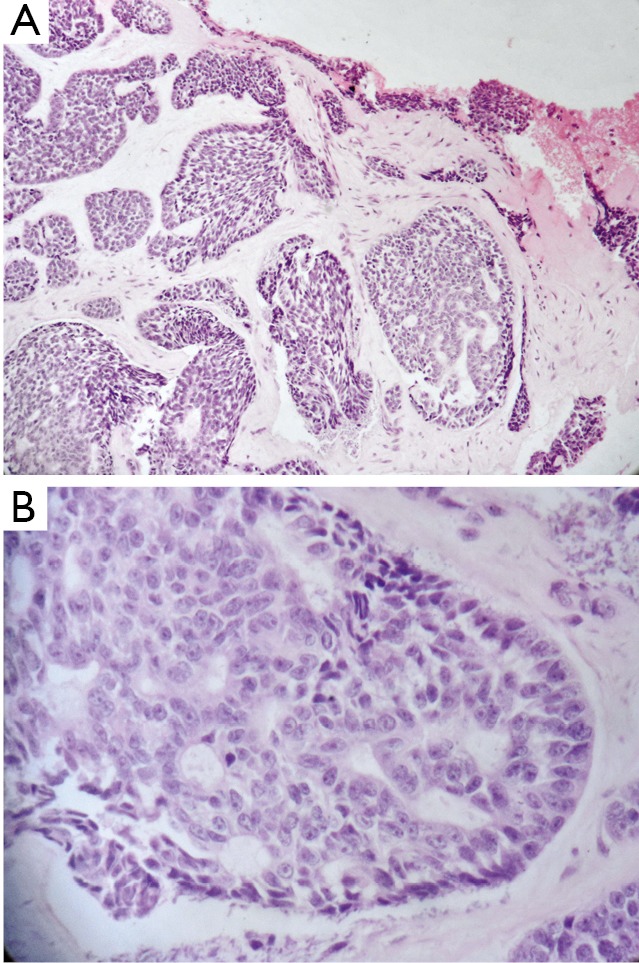
Micrographs of the ACC stained with hematoxylin and eosin. A. Prominent solid and microcystic patterns of the ACC with the ulcer margins (200×); B. Details of the ACC, showing a cribriform pattern and its tumor cells (400×).

Upon admission, malignant mesenchymal or odontogenic neoplasia was suspected. An incisional biopsy was then performed under local anesthesia. The histopathological and immunohistochemical features were diagnostic of ACC. Microscopic examination showed prominent solid, microcystic, and focally tubular glandular patterns of growth, as well as numerous cells with myoepithelial characteristics and a few luminal cells. The histopathological features were similar to those of the solid subtype of salivary gland ACC (**Figure 2**). A panel of antibodies (Dako, USA) was used for immunohistochemical evaluation, namely, those for cytokeratins (CK5, 05/16B4; CK7, OV-TL12/30), smooth muscle actin (SMA, 1A4), p63 (4A4), and Ki-67 (MIB-1).

The patient underwent left total maxillectomy, followed by buccal fat pad flap reconstruction. Histopathological analysis of the resected specimen showed similar features with those of the incisional biopsy. The surgical margins revealed no residual carcinomas.

Extensive clinico-radiological investigations were performed to identify potential metastasis. Chest radiography, as well as computerized tomography of the head and neck regions, was conducted. No metastatic lesions or other primary neoplasms were detected. The patient remained in good health after  25 months of follow-up, with no evidence of local recurrence.

## Discussion

Carcinomas of the maxillary gingiva are relatively common but easily neglected by patients. Such tumors differ from those that originate from the maxillary sinus and have secondary extensions to the gingiva. Radiography of the paranasal sinuses aids differential diagnosis and allows for the careful evaluation of the extent of bone involvement. Gingival carcinomas in the maxilla usually invade the maxillary sinus. These carcinomas may extend onto the palate or the tonsillar pillar.

**Figure 3:**
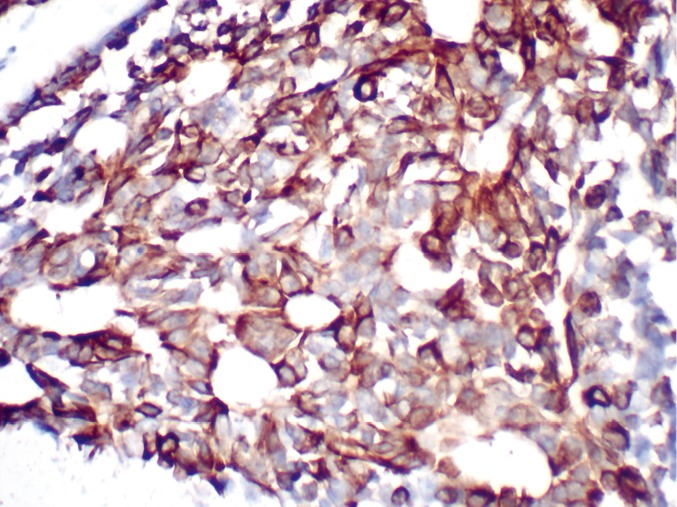
Immunohistochemical analysis showing intense CK7 expression.

**Figure 4:**
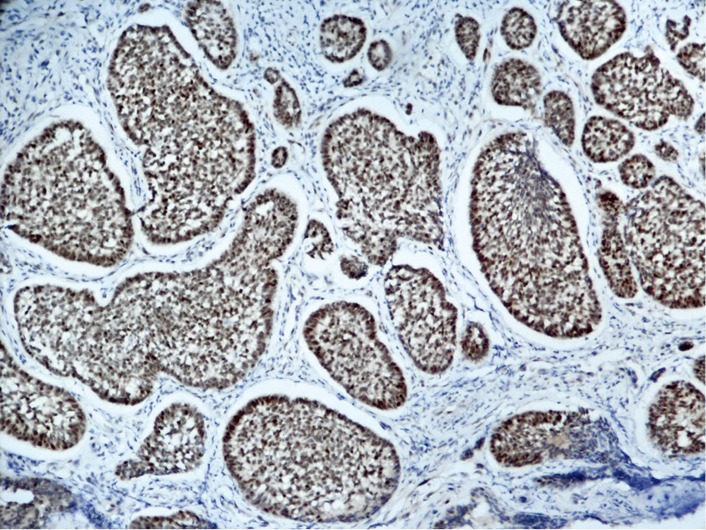
Strong positive p63 expression in myoepithelial cells.

ACC is one of the most common tumors in the salivary glands; it mainly involves the palate but rarely affects the gingiva^5^. Patients are typically presented with a slowly enlarging mass, which could become considerably large because of its indolent growth pattern. The mass is usually painless, although its bone invasion or perineural spread could cause pain or hypoesthesia. Most ACCs are submucosal, appearing as smooth, domed swellings without overlying ulceration. Swelling may be unencapsulated, but it is frequently well circumscribed. Their insidiously infiltrative growth pattern could be misleading. Three histological subtypes of ACC are known, namely, the cribriform, tubular, and solid subtypes. All subtypes may occur either separately or together in the same tumor; the solid subtype is the most aggressive among the three^3^. Immunohistochemistry helped establish the diagnosis for the current case. CK5 and CK7 (**Figure 3**) were intensely stained in the luminal cells, whereas both were scarce in the myoepithelium-like cells. The myoepithelial component was deeply stained with p63 (**Figure 4**) and SMA. The labeling index of Ki-67 was higher than 30%. This index was calculated after analyzing approximately 1,000 cells in five high-power fields. A high Ki-67 index and the presence of more than 50% of the solid areas may indicate an aggressive clinical course^2^^,^^6^. The incidence of cervical lymphatic metastasis was low; it was approximately 8% at presentation but eventually decreased to 7%^7^.

Most, if not all, patients with long-term ACC eventually develop distant metastases, mainly in the lungs and bone, despite the local control of the tumor. The occurrence of bone metastasis usually corresponds to rapid tumor dissemination and death of the patient, whereas lung metastases demonstrate a less aggressive clinical course^8^.

In this case, the central salivary gland tumors should be included in the differential diagnosis. These tumors develop within the mandible or maxilla and can extend to the more usual locations. Most central salivary gland tumors of the jaw involve the mandible, which account for approximately 65% of intraosseous ACC^6^. The pathogenesis of central salivary gland tumors may include the neoplastic transformation of the epithelial linings of odontogenic cysts, the metaplasia of the epithelial rests of Malassez, or the presence of ectopic salivary gland tissue^8^^-^^10^.
